# Prognostic Insights in Feline Mammary Carcinomas: Clinicopathological Factors and the Proposal of a New Staging System

**DOI:** 10.3390/ani15060779

**Published:** 2025-03-10

**Authors:** Mónica Monteiro, Gonçalo Petrucci, Felisbina L. Queiroga

**Affiliations:** 1 Department of Veterinary Sciences, University of Trás-os-Montes and Alto Douro, 4150-562 Porto, Portugal; monicalilianasmonteiro@gmail.com; 2 OneVet Group, Hospital Veterinário do Porto, 4150-562 Porto, Portugal; goncalo.petrucci@onevetgroup.pt; 3 Department of Animal and Veterinary Sciences, University Institute for Health Sciences, Cooperativa de Ensino Superior Politécnico e Universitário (CESPU), Rua Central de Gandra, 1317, 4585-166 Gandra, Portugal; 4 Animal and Veterinary Research Center (CECAV), University of Trás-os-Montes and Alto Douro, 5001-801 Vila Real, Portugal; 5 Associate Laboratory for Animal and Veterinary Sciences (AL4AnimalS), 1300-477 Lisboa, Portugal

**Keywords:** mammary carcinomas, staging, prognosis, TNM system, survival analysis, Cox regression

## Abstract

Feline mammary carcinoma (FMC) is an aggressive neoplasm with a poor prognosis. Clinical staging is crucial for risk assessment, yet the current WHO system lacks granularity, particularly in Stage III. Aligning this system with human breast cancer staging has the potential to improve prognostic accuracy. Additionally, prognostic factors such as tumor size, ulceration, lymph node metastasis, and lymphovascular invasion require further evaluation. This study retrospectively analyzed 75 female cats with FMC to assess the prognostic impact of clinicopathological factors and evaluate a novel staging system (new staging) adapted from the *AJCC Cancer Staging Manual*. Survival analyses included disease-free interval (DFI), and overall survival (OS). Tumor size >3 cm (*p* < 0.001), ulceration (*p* = 0.010), lymphovascular invasion (*p* < 0.001), lymph node metastasis (*p* < 0.001), WHO and new staging (*p* < 0.001) were significantly associated with shorter survival. The new staging refined Stage III into III_A_ (T_3_N_0_M_0_), III_B_ (T_4_N_0_M_0_), and III_C_ (AnyTN_1_M_0_), improved prognostic differentiation. Stage III_C_ cases had the worst survival (*p* < 0.001). Multivariate survival analysis identified lymphovascular invasion (HR = 2.834, 95% CI: 1.546–5.195, *p* = 0.001), histological Grade II (HR = 5.013, 95% CI: 1.122–22.397, *p* = 0.035) and III (HR = 9.894, 95% CI: 2.195–44.594, *p* = 0.003) and skin ulceration (HR = 2.462, 95% CI: 1.256–4.825, *p* = 0.009). These findings support the prognostic relevance of key clinicopathological factors in FMC and highlight the advantages of a refined TNM-based staging system, which may enhance risk stratification and therapeutic decision-making in veterinary oncology.

## Simple Summary

Mammary carcinoma is a major health concern in female cats, yet the lack of standardized prognostic tools limits accurate risk stratification and treatment planning. This study evaluates the applicability of a human-based breast cancer staging system (*AJCC Cancer Staging Manual*) in feline oncology, aiming to refine prognostic assessments. Through a retrospective analysis of 75 cats, we identified ulceration, tumor size >3 cm, and lymph node metastasis as significant predictors of poorer survival. Although inflammatory carcinomas were not observed, we propose integrating a T_4_ category into the classification system, considering cats’ susceptibility to infections and inflammatory complications. The new staging system improved risk differentiation by subdividing Stage III into III_A_ (T_3_N_0_M_0_), III_B_ (T_4_N_0_M_0_), and III_C_ (AnyTN_1_M_0_), allowing better treatment stratification. These findings emphasize the need for harmonized, standardized reporting protocols to enhance consistency across studies and foster international, interdisciplinary collaboration. By adapting human oncology frameworks to veterinary medicine, this study provides a valuable tool to improve prognostic accuracy and optimize therapeutic strategies. Ultimately, these insights advance the scientific understanding of feline mammary carcinoma, support evidence-based clinical decision-making, and lay the foundation for future research in veterinary oncology.

## 1. Introduction

Mammary gland carcinomas represent a significant concern in veterinary clinical practice, particularly in feline patients. Recent cross-species analyses comparing mammary tumors in dogs and cats have highlighted critical differences in tumor incidence and malignancy. Notably, cats are approximately twice as likely as dogs to develop mammary tumors and five times more likely to develop malignant forms [1]. Epidemiological studies have reported a wide range of incidence statistics (from 13 to 104 per 100,000 cats per year), reflecting differences in diagnostic methodologies, clinical practices, and the cultural and demographic contexts of the respective countries from which the data were collected [2,3,4,5]. Despite this variability, feline mammary carcinomas (FMCs) consistently rank among the three most prevalent neoplasms in cats and are a leading cause of oncological death [6,7,8]. The literature identified key risk factors for FMC development, with age, breed, and hormonal influences being the most prominent. Middle-aged to older female cats, particularly purebred, are at higher risk, especially those sterilized late in life or those with a history of frequent progestin use [2,3,4,6,9,10,11]. The aggressive biological behavior, characterized by local invasion and a marked propensity for metastasis to distant organs [12], complicates clinical management and contributes to a poorer prognosis. Additionally, the elevated mortality rate and existing gaps in the scientific understanding of this disease present ongoing challenges in both veterinary practice and oncological research [13,14,15,16,17].

In recent years, there has been renewed interest in elucidating the factors that influence the prognosis of FMCs [12,13,18]. Understanding these factors is crucial for improving clinical decision-making and fostering effective communication with guardians [14]. However, challenges in early detection and the lack of standardized treatment protocols hinder optimal outcomes. Several prognostic markers, including tumor size, histological grade, and lymph node involvement, have been identified as key determinants of survival and recurrence [13]. For instance, previous studies have shown that smaller tumor sizes (<2 cm) are consistently associated with better outcomes, with some cases achieving remission through surgical intervention alone (particularly radical surgery), resulting in an average survival of 3 years [9]. In contrast, literature reports indicate that tumors larger than 3 cm are associated with significantly shorter disease-free survival (4 to 6 months), even after surgical intervention [19,20,21,22]. Lymph node metastasis is another independent factor that negatively affects prognosis [19,21,23]. Furthermore, lymphatic invasion has been shown to reliably predict lymph node metastasis, further correlating with reduced survival times [12,15,20,21,24]. However, other factors, such as the role of cutaneous ulceration and clinical staging, remain poorly understood, limiting the ability to tailor treatment strategies for individual patients.

In comparative oncology, the similarities between feline mammary carcinomas and human breast cancer offer unique opportunities for translational research [25,26,27,28,29,30,31]. Both species exhibit parallels in clinicopathological, anatomical, and epidemiological factors [28,32,33], yet disparities remain in clinical approaches. The prognosis of human breast cancer has advanced significantly, owing to the concerted efforts of multidisciplinary teams and the establishment of internationally recognized clinical guidelines. These frameworks facilitate the dissemination of research findings and ensure that advancements in clinical care are accessible to healthcare providers globally [34,35,36]. Additionally, the periodic revision of the TNM staging system, in response to emerging clinical evidence, not only refines diagnostic accuracy but also enhances the effectiveness of treatment protocols, ultimately improving patient outcomes. In contrast, similar progress has been slower in veterinary oncology [17,37], partly due to retrospective study designs, incomplete datasets, and inconsistent terminologies [13,38,39].

The tumor–node–metastasis (TNM) staging system is widely used in human and veterinary oncology [39,40,41]. However, its application in veterinary medicine presents several limitations. The current TNM classification for FMCs, adapted from the World Health Organization (WHO) [40], lacks sufficient granularity to distinguish between tumors with different levels of aggressiveness. While the system has undergone slight adaptations over the past 44 years [39,42], it has shown limitations in accurately capturing disease progression. Notably, large tumors (>3 cm) and cases with lymph node metastases are grouped under Stage III despite having distinct prognostic implications. Moreover, the absence of the T_4_ category (which accounts for tumors with extension to the thoracic or abdominal wall or involving the skin [40], typically associated with poor clinical outcomes) is a significant gap [39,43]. This lacuna in the classification system was addressed by Chocteau et al. (2019) [15], who proposed the addition of a T_4_ category for ulcerated FMCs, thereby underscoring the prognostic significance, improving clinical decision-making and ultimately enhancing patient outcomes.

To the best of our knowledge, no study has evaluated the impact of a more refined classification system on the clinical staging of FMCs. Aligning this system with human breast cancer staging aims to improve prognostic accuracy, survival predictions, and treatment customization. Therefore, we selected the seventh edition of the *AJCC Cancer Staging Manual*, as a reference [41]. This system prioritizes anatomical and pathological features, minimizing reliance on molecular diagnostics, which are often inaccessible in veterinary medicine [44,45]. Additionally, it offers sufficient granularity for staging while remaining practical for routine clinical use [46,47,48]. Tumors are classified into Stages I to IV, reflecting increasing malignancy [40,41,49].

The current feline system lacks precision, grouping all cases with large tumors (>3 cm) or lymph node involvement into Stage III, despite their distinct prognostic implications [9,42,50]. To address this, we propose a novel clinical staging system (new staging (NS)) with three subcategories: Stage III_A_ (T_3_N_0_M_0_) for tumors larger than 3 cm without lymph node involvement, Stage III_B_ (T_4_N_0_M_0_) for tumors of any size with direct extension to surrounding tissues (e.g., chest wall, abdominal wall, or skin, including ulceration), and Stage III_C_ (AnyTN_1_M_0_) for cases with regional lymph node metastases.

This study aimed to investigate the impact of clinicopathological parameters and treatment approaches on the prognosis of feline mammary carcinoma (FMC). Additionally, it assessed the potential advantages of a novel clinical staging system adapted from the seventh edition of the *AJCC Cancer Staging Manual*, in comparison with the previously used classification. While molecular markers and genetic variations may influence FMC prognosis and treatment response, this study did not focus on these factors. Through retrospective analysis and survival evaluation (disease-free time and overall survival), we aimed to determine whether this refined staging approach provides better prognostic stratification and clinical applicability. Implementing this system could bridge existing gaps in veterinary oncology, enabling more effective treatment strategies and ultimately improving patient outcomes.

## 2. Materials and Methods

### 2.1. Case Selection

A retrospective analysis was conducted at Onevet Hospital Veterinário do Porto. All included cats underwent surgical excision of the primary tumor, and intact females were concurrently subjected to ovariohysterectomy. Inclusion criteria required a histopathological diagnosis of mammary carcinoma, while exclusion criteria encompassed incomplete diagnostic, treatment, or follow-up records.

### 2.2. Data Collection

Clinical records were retrieved from the hospital database, including breed, age, reproductive status at mastectomy, lesion number and location, ulceration status, tumor size, lymphatic involvement, distant metastases, WHO staging, and surgical details. The histopathological report furnished data about the histological degree (classification adapted from Elston and Ellis [51]), the histological type, the presence of lymphovascular invasion, and the presence of lymph node metastasis. The histological classification of the tumors was carried out following the World Health Organization (WHO) classification [52].

**Figure 1:**
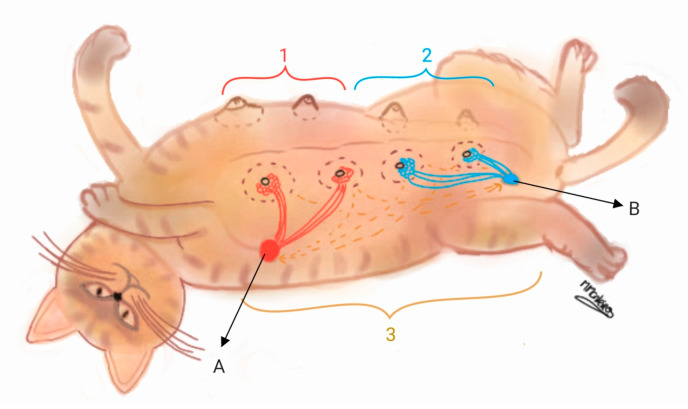
Mammary carcinoma anatomical locations considered for this study. 1 = Thoracic location (T1—cranial thoracic mammary gland; T2—caudal thoracic mammary gland); 2 = abdominal location (A1—cranial abdominal mammary gland; A2—caudal abdominal mammary gland); 3 = simultaneous thoracic and abdominal; A = axillary lymph node; B = inguinal superficial lymph node. This is an original illustration by Mónica Monteiro.

Considering the available data and the lymphatic drainage pattern of cats [53,54], three locations were identified for the carcinomas: 1. Thoracic location—in the vicinity of the thoracic mammary glands (T1 and T2); 2. Abdominal location—in the area encompassed by the abdominal mammary glands (A1 and A2); 3. Simultaneous thoracic and abdominal location (Figure 1). It is important to note that this classification was employed exclusively for ipsilateral lesions. The surgical approach was categorized as partial mastectomy (removal of affected gland/s); unilateral radical mastectomy (removal of the entire mammary chain); and bilateral radical mastectomy, either in one stage or two staged procedures.

### 2.3. Current Clinical Staging System

Clinical staging was first performed under the modified WHO system [40,42]. Tumor size was determined as the largest diameter of the visible lesion into three categories: T_1_ (less than 2 cm in diameter), T_2_ (between 2 and 3 cm), and T_3_ (more than 3 cm) [40]. The involvement of regional lymph nodes and the presence of distant metastases were evaluated through physical examination and imaging techniques, including thoracic X-rays (3 projections), whole-body computed tomography, and abdominal ultrasound. All lymph nodes were subjected to cytological and/or histopathological confirmation, and the specific lymph node affected (axillary or superficial inguinal) was broken down in the study. Whenever feasible, and without worsening the animal’s clinical condition, distant metastases were also confirmed using the techniques described above. However, some cats with pulmonary nodules were considered to have metastases based solely on the imaging findings and symptomatology, as described in other studies [16,24].

### 2.4. Proposal for a New Staging System

To refine prognostic stratification, we proposed a novel clinical staging system (new staging (NS)), adapting the seventh edition of the *AJCC Cancer Staging Manual* [41]. This modification aimed to address the limitations of the WHO system, particularly the lack of differentiation within Stage III [9,15,39].

In the NS, Stages I, II, and IV remain unchanged, while Stage III is subdivided into three categories (Table 1).

### 2.5. Follow-Up and Survival Endpoints

Follow-up was performed for at least one-year post-mastectomy, with appointments scheduled every three months during the first year and every six months thereafter. In cases where the last consultation had occurred more than six months before, owners were contacted to obtain updated clinical information. Local recurrence was defined as the presence of a population of neoplastic cells, confirmed by cytology or histopathology, in the eminences of the surgical scar of the previously removed tumor [55,56,57,58].

The disease-free interval (DFI) was defined as the interval between the surgical excision of the primary tumor and the occurrence of either local recurrence or distant metastatic disease. Overall survival (OS) was defined as the time between mastectomy and death, regardless of the cause.

### 2.6. Statistical Analysis

The statistical analysis was conducted using the Statistical Package for the Social Sciences (SPSS) software, version 29.0. Categorical variables were presented as absolute and relative frequencies, while numerical variables were expressed as mean ± standard deviation, median, and range. Comparisons of categorical variables were performed using Pearson’s chi-squared test, with Fisher’s exact test applied when appropriate.

Survival curves were generated using the Kaplan–Meier method, and survival rates were compared using the log-rank test. Two temporal cut-off points, 500 and 1000 days, were used for survival analysis. Cats that were alive at the end of the study or lost to follow-up were censored at the last recorded contact.

The impact of the WHO stage and the new staging on study outcomes was also assessed using an unadjusted (univariable) Cox proportional hazards regression model. Multivariate Cox regression was conducted using the forward conditional method, in which variables were progressively introduced into the model based on the statistical significance criterion (*p* < 0.05). A *p* value <0.05 was considered statistically significant.

## 3. Results

### 3.1. Epidemiological Data

Seventy-five female cats met the inclusion and were included in the study. Regarding breed, the majority (*n* = 66; 88%) of cats were European domestic short hair, followed by Persian (*n* = 4; 5.33%), Somali (*n* = 3; 4%), Siamese (*n* = 1; 1.33%), and Norwegian Forest (*n* = 1; 1.33%). The mean age of the cohort was 11 ± 3 years (range 1–18.5, and median of 11 years), and for the survival analysis, this variable was dichotomized into the first category, comprising cats aged ≤11 years (*n* = 41/75; 55%), and the second, comprising cats aged >11 years (*n* = 34/75; 45%). At the time of surgery, forty-four cats were spayed (59%) and 31 (41%) were intact.

As previously stated in Section 2, all cats underwent surgical removal of the primary tumor. Approximately half of the subjects underwent partial mastectomy (50.7%, *n* = 38), while 36.0% (*n* = 27) underwent unilateral radical mastectomy and 13.3% (*n* = 10) underwent bilateral radical mastectomy. According to the records, 37.3% of the population (*n* = 28/75) received adjuvant treatment following mastectomy. Of these, 71.4% (20/28) were administered with metronomic chemotherapy, 25.0% (*n* = 7/28) underwent a protocol of metronomic chemotherapy in conjunction with immunotherapy, and 3.6% (1/28) received only doxorubicin.

Follow-up information was available for all of the animals included in the study. The records revealed a high percentage (62.7%; *n* = 47/75) of animals with disease recurrence throughout the study. Local recurrence was confirmed in 5 of the 75 cats (6.7%). In turn, distant metastases were documented in 30.7% of the population (*n* = 23/75). Nevertheless, 19 (25.3%) of the cats experienced both local and distant disease recurrence. In the cohort of twenty-three cats with distant metastases, two had liver metastases, one had multiple metastasis (affecting the muscles, bones, lungs, bladder, sciatic nerve, and iliac, inguinal, and popliteal lymph nodes), and the remaining cats had lung metastases. Despite the research team’s diligent efforts, 5 of the 75 cats were unfortunately lost to follow-up during the study. For these cases, the date of the last contact was used as a censoring condition when calculating survival intervals. By the end of the work, 74.7% (*n* = 56/75) of the population had died, while the remaining 25.3% (*n* = 19/75) was distributed between the 14 cats that remained alive and the 5 that were lost to follow-up. Of the 56 cats that died, 41 (73%) succumbed to the progression of malignant mammary neoplasia.

### 3.2. Tumor Characteristics

The clinicopathological characteristics of the 75 mammary carcinomas are summarized in Table 2. The data set encompassed all the cats, except the anatomic location variable, for which information was only available for 71 cases.

### 3.3. Clinical Staging System

All cats (*n* = 75) were classified according to the World Health Organization (WHO) staging system, with 30.7% (*n =* 23) in Stage I, 13.3% (*n =* 10) in Stage II, 53.3% (*n =* 40) in Stage III, and 2.7% (*n =* 2) in Stage IV. When reclassified using the new staging (NS) system, the distribution changed, with 28.0% (*n =* 21) in Stage I, 10.7% (*n =* 8) in Stage II, and the remaining cases distributed across subcategories III_A_ (6.7%, *n =* 5), III_B_ (8.0%, *n =* 6), III_C_ (44.0%, *n =* 33), and IV (2.7%, *n =* 2). Animals previously classified as WHO Stage I or II, in which the presence of T_4_ is acknowledged, are now reclassified as Stage III_B_ in the new system. The differences between the two classification methods were statistically significant (*p* < 0.001).

### 3.4. Survival Analyses

The mean DFI observed in the study population was 442 ± 426 days, with a median of 296 days (range: 20–1855 days). For OS, the mean was 510 ± 422 days, with a median of 361 days (range: 30–1855 days). Several factors significantly and negatively impacted both endpoints, including the presence of skin ulceration, larger tumor size, lymph node metastasis, high histological grade, and lymphovascular invasion (Table 3). Animals diagnosed with metastatic disease at initial presentation had significantly shorter survival times.

**Figure 2:**
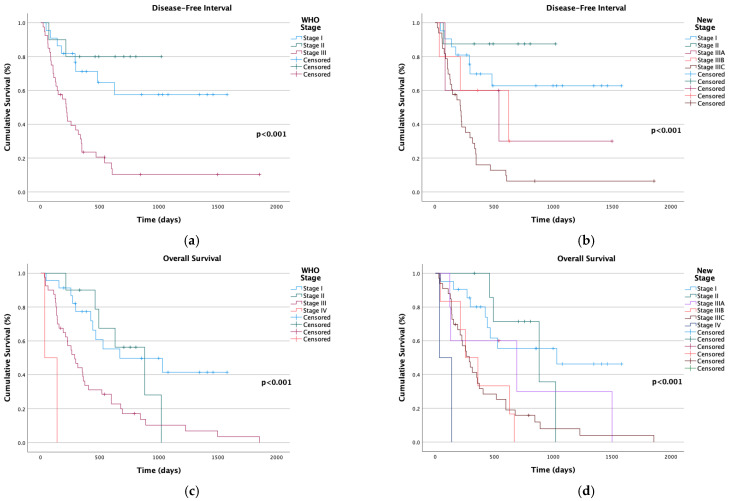
Kaplan–Meier survival curves for disease-free interval (DFI) and overall survival (OS) in female cats with mammary carcinoma, categorized by clinical staging methodology. Tick marks indicate censored cases. (**a**) Disease-free interval (DFI) stratified by the World Health Organization (WHO) staging system. Statistically significant differences were observed (*p* < 0.001); (**b**) disease-free interval (DFI) stratified by the new staging system. Median DFI values were 541 days for Stage III_A_, 624 days for Stage III_B_, and 215 days for Stage III_C_. Differences were statistically significant (*p* < 0.001); (**c**) overall survival (OS) stratified by the WHO staging system, showing statistically significant differences (*p* < 0.001); (**d**) overall survival (OS) stratified by the new staging system. Median OS values were 691 days for Stage III_A_, 255 days for Stage III_B_, and 288 days for Stage III_C_. Differences were statistically significant (*p* < 0.001).

Staging was significantly correlated with both DFI and OS, regardless of whether the WHO or new staging system was used (*p* < 0.001). In the WHO system, cases classified as Stages I and II had longer DFI compared with Stage III (Figure 2a). Under the new staging system, Stages I and II showed a slight increase in average DFI compared with their WHO counterparts (Stage I: 1037 vs. 1079 days; Stage II: 846 vs. 903 days). Stage III in the WHO system closely corresponded to Stage III_C_ in the new staging system, with similar median DFI values. In the new staging system, only 12.8% of Stage III_C_ cases remained disease-free at 500 days. At 1000 days, 30% of Stage III_A_ cases were disease-free, whereas no cases classified as III_B_ reached this time point (Figure 2b). Clinical staging was identified as a parameter with a highly significant impact on predicting overall survival. Cases classified as Stage IV, regardless of the classification approach, showed a propensity to die before the first cut-off. The highest values were observed for Stages I and II at both 500 days and 1000 days, although the new stage suggests slightly higher values (Figure 2c,d). The results obtained for Stage III are analogous to those of the new III_C_, with both demonstrating a median value of 288 days. Notwithstanding the elevated degree of malignancy, the subjects in this stage attained the 1000-day milestone, a benchmark that has not been achieved by those categorized as Stages III_A_ and III_B_ (Figure 2d).

To assess the risk associated with staging classifications in terms of disease recurrence and mortality, a univariable Cox regression analysis was performed (Table 4).

A Cox proportional hazards regression model was performed using the forward conditional method to identify independent prognostic factors for survival. The final model retained lymphovascular invasion (HR = 2.834, 95% CI: 1.546–5.195, *p* = 0.001), histological Grade II (HR = 5.013, 95% CI: 1.122–22.397, *p* = 0.035) and Grade III (HR = 9.894, 95% CI: 2.195–44.594, *p* = 0.003), and ulceration (HR = 2.462, 95% CI: 1.256–4.825, *p* = 0.009) as significant independent predictors of overall survival.

## 4. Discussion

This study aimed to investigate the prognostic role of clinical–pathological variables and to assess the applicability of a new staging system adapted from the seventh edition of the *AJCC Cancer Staging Manual*. It is hypothesized that by aligning clinical staging with that employed in women, the prognostic value of clinical staging in veterinary clinical practice can be enhanced, thereby facilitating advancements in translational research. The findings underscore the importance of understanding these factors for improving veterinary oncology diagnosis, prognosis, and treatment strategies.

Epidemiological evidence has identified breed as a potential risk factor for the development of mammary neoplasia [6,9,13]. In this study, the most prevalent breed was the Common European, comprising nearly all of the observed individuals. However, the impact of breed on survival was not evaluated due to the limited number of specimens from other breeds, which precluded obtaining statistically significant results.

The mean age of the felines was 11 years, which is consistent with the range of 10 to 12 years reported in analogous studies [2,4,6,59,60,61]. Notably, disease-free intervals (DFIs) were higher in cats over 11 years of age, possibly due to the presence of younger cats with advanced carcinomas and aggressive histological types, such as micropapillary carcinomas [21,60,62,63]. Furthermore, the literature highlights the protective role of early sterilization practices in reducing the risk of mammary neoplasia. The most important benefit is reported when ovariohysterectomy is performed before six months of age, with the protective effect diminishing markedly by 24 months and becoming negligible thereafter [11,64]. At the time of tumor excision, more than half of the studied population had undergone ovariohysterectomy. However, the lack of precise data regarding the timing of sterilization relative to each cat’s reproductive cycle constrained the ability to assess its role as a prophylactic measure against mammary neoplasia. As noted by Pickard Price et al. (2023) [4], the association between neuter status and mammary tumor diagnosis remains inconclusive, with existing literature presenting contradictory findings [4,14,65]. This underscores the complexity of evaluating this variable, which is further compounded by demographic disparities and inconsistent adherence to sterilization recommendations among pet owners.

Skin ulceration was documented in 18.7% of the cats and demonstrated a statistically significant association with DFI and OS, for which average values of 7, and 9 months were obtained, respectively. These findings align with previous studies, underscoring the poor prognosis linked to skin ulceration [15,23,66,67], which reflects an advanced disease stage [68,69,70], an elevated risk of progression [24,71], and a higher likelihood of local recurrence [66,72]. Our findings demonstrate that ulceration functions as an independent predictor of poor OS, signifying the potential benefit of more aggressive treatment strategies for patients with ulcerated tumors. This assertion is consistent with the current literature on melanoma, where ulceration is utilized for staging to inform clinical decisions. The presence of ulceration has also been linked to a more immunosuppressive tumor microenvironment, which may influence responses to therapy [69,70,73,74]. Given its strong prognostic value, skin ulceration should be recognized as a malignant trait and incorporated into the TNM staging system alongside other critical parameters [75,76,77,78]. Consequently, patients with the new Stage III_B_ disease may require more intensive adjuvant therapy, such as immunotherapy or chemotherapy, as well as closer follow-up schedules to detect recurrence early.

Histopathological assessment remains the gold standard for diagnosing mammary carcinomas [79,80,81]. The most prevalent histological type in this study was tubulopapillary carcinoma, though its prognostic significance remains unclear, as the literature is divided on whether this characteristic predicts survival [13,21,82,83]. A greater proportion of high-grade carcinomas, as classified by Elston and Ellis (1991) [51], was observed, significantly reducing survival estimates. High-grade carcinomas, which reflect increased differentiation, proliferation, and cellular atypia, are associated with poorer outcomes [84]. As documented in prior studies [12,21,82,85], surgical removal of low-grade neoplasms generally results in more stable post-operative survival rates, while Grade III carcinomas—characterized by rapid proliferation, often with incomplete margins, necrosis, or peritumoral inflammation—have survival rates below 30% within one year of diagnosis [15,18,76,80]. Interestingly, none of the cats with Grade I carcinoma died due to the progression of the neoplastic disease. Multivariable Cox proportional hazard analysis corroborated this strong association between high-grade and heightened mortality risk. Specifically, patients with Grade II tumors exhibited a significantly elevated hazard of death (HR = 5.013, *p* = 0.035), and those with Grade III tumors showed an even greater risk (HR = 9.894, *p* = 0.003) compared with Grade I tumor counterparts. Accordingly, patients with high-grade tumors may benefit from more intensive multimodal treatment approaches, including chemotherapy, targeted therapy, or closer surveillance to improve outcomes.

At the time of diagnosis, 45% of the cats already presented with locally advanced disease. Regional lymph node invasion, when confirmed via histopathological analysis, is a highly reliable prognostic factor and is independently associated with overall survival (OS) [13,21]. In our analysis, lymph node invasion significantly predicted DFI and OS (<0.001). Consequently, only 12.8% of the population remained disease-free at 500 days. For the same period, it was estimated that 28% would survive.

Lymphovascular invasion was documented in 36% of the carcinomas assessed. This parameter is particularly valuable when regional lymph node sampling is unavailable, as it allows for the assessment of lymphatic dissemination [15,86,87]. Lymphatic invasion thus serves as a strong predictor of lymph node metastases, even more so than in dogs, due to advancements in the histological evaluation of lymphatic emboli using immunolabelling techniques targeting the endothelial cells of lymphatic vessels with an anti-LMO2 antibody [12]. This feature is also considered predictive of nodal metastasis and local recurrence in women with breast cancer [88,89]. In our study, the presence of lymphovascular invasion resulted in extremely low DFI and OS values, with no parameter exceeding an average of nine months. This significant impact on survival estimation is widely accepted by veterinary oncologists, with some even advocating for its inclusion in the parameters that determine histological grade [12,13,15,20,21,23,71,90,91,92]. In contrast to tumor size, Chocteau et al. (2021) [93] reported that animals with nodal involvement and/or lymphovascular invasion continue to face a high long-term risk of cancer-related death due to distant metastases. Additionally, lymphovascular invasion remained statistically significant, reinforcing its role as an independent prognostic factor.

Surgery remains the primary treatment for mammary carcinoma in cats, and more extensive surgical interventions have been associated with longer disease-free and overall survival periods. In particular, bilateral mastectomy has been linked to superior progression-free and tumor-specific survival [24,42,68,94], whereas more conservative approaches have been identified as a risk factor for recurrence or local metastasis [24,95]. However, in our study, no statistically significant differences were observed between surgical approaches and survival outcomes. This finding must be interpreted with caution, as several factors may have influenced the results. One key limitation is the small number of cats that underwent bilateral mastectomy in our cohort (10 out of 72 cases), which may have reduced the statistical power necessary to detect significant differences. Additionally, the use of adjuvant treatments, such as chemotherapy, was not standardized across the study population, introducing potential variability in survival outcomes. Despite the lack of statistical significance, the trend observed in our data aligns with previous reports, reinforcing the rationale for considering radical bilateral mastectomy as the standard approach for treating cats with mammary carcinoma.

A further objective of this study was to examine the prognostic value of a novel clinical staging system for feline mammary carcinoma, to update a staging system that is more aligned with those utilized in human breast cancer. The TNM system, which classifies tumors based on anatomical extension, enables the grouping of individuals with similar prognoses [96].

Staging is strongly correlated with survival, whether conducted under WHO recommendations or the new system. Metastatic mammary carcinomas (Stage IV) have a guarded prognosis, and, especially in this study, cats with distant metastases survived no more than three months on average. However, a study conducted by Petrucci et al. (2021) [16], which evaluated the largest number of cats in this condition, suggests that some cats manage to survive beyond six months, particularly asymptomatic cats, which tend to have higher survival rates. Therefore, investment in palliative surgery and adjuvant treatments is essential to improve the quality of life for these animals. In human medicine, the approach is also palliative, focusing on symptomatic support through chemotherapy, hormone therapy, targeted therapies, and radiotherapy [34].

Stages I and II are primarily determined by tumor size and are considered biologically locally invasive [41,97,98]. Tumor size is widely regarded as one of the most influential prognostic factors [12,15,22,23,94], as it reflects the number of cell divisions, accumulation of mutations, and increased susceptibility to progression into more aggressive behavior [39]. In this study, higher tumor size was statistically significantly associated with DFI and OS. Interestingly, neoplasms with diameters between 2–3 cm showed the best survival outcomes in the analysis [DFI (mean): T_1_|T_2_—21|42 months; OS (mean): T_1_|T_2_—23|30 months]. A possible explanation for these unexpected results is that the T_2_ category was the least prevalent (*n* = 16/75), suggesting that further studies with larger sample sizes are needed to reach more definitive conclusions. Recent research by Chocteau et al. (2021) [93] has determined that, after six months post-diagnosis, tumor size no longer holds significant prognostic value. However, as the aim of this study was not to evaluate conditional survival and more conservative surgeries were performed, this effect was not reflected in the cumulative survival percentages. In women, the Surveillance, Epidemiology, and End Results (SEER) database estimates that 99% of patients diagnosed at these stages survive the first five years [99]. In comparison, female cats have two-year survival rates ranging from 28% to 60%, depending on the stage and cause of death. This difference is justifiable due to well-organized screening programs for women and the interdisciplinary nature of therapeutic decisions, which are informed by clinical evidence, randomized studies, expert committees, and protocol guidelines [34,35,98,100].

Stage III is recognized as a regional stage [41]. The values obtained for DFI, and overall survival (OS) were similar to those reported in recent studies evaluating the prognostic value of a new histological staging system [15]. When subcategorized into the New Stage system proposed in this study, cats in Stage III_A_ (T_3_N_0_M_0_) exhibited overall cumulative survival rates very similar to those in Stage II at the 500-day mark. For Stages III_B_ and III_C_, it was not immediately clear whether nodal invasion or skin ulceration had more influence on survival. However, when cumulative survival was assessed, considerable differences were observed at 500 days, with a disease-free interval of 60% for Stage III_B_ versus 12.8% for Stage III_C_, and overall survival of 33.3% for Stage III_B_ versus 28.5% for Stage III_C_. In the long term, these differences became even more pronounced, with only a small percentage of cats in Stage III_C_ surviving beyond 1000 days, while no cats with ulceration in Stage III_B_ survived. Univariate analysis revealed that both the WHO stage and the New Staging system had a highly significant impact on DFI and OS. However, the New Stage system demonstrated superior prognostic value, particularly in distinguishing Stage III_C_, which was associated with significantly poorer outcomes in both DFI and OS compared with other stages. This refined classification provided a more precise stratification of risk, emphasizing the aggressive nature of Stage III_C_. Furthermore, Stage IV exhibited the worst prognosis, with a high hazard ratio for OS (HR = 23.171), highlighting the critical need for early detection and timely intervention.

Recurrence of the disease is common in cats, even after mastectomy. The definition of local recurrence is not consensual either among studies carried out on feline mammary carcinomas [24,55,57,101] or those carried out in the context of women’s breast cancer, which also determines four distinct types of local recurrence [102]. Following the definition used in the methodology of this experimental study, 30.7% of the population exhibited local recurrence, 56% presented with distant metastases, and 21% of the cats showed both local and distant recurrence. These findings are consistent with the results described by Gemignani et al. (2018) [24] and Soares et al. (2016) [71]. Given that distant metastasis generally displays a worse immunophenotype than the corresponding primary tumor due to the adaptive response to the microenvironment of different tissues [71,103], early identification is crucial in the clinical follow-up of these animals. The occurrence of local recurrence and distant metastases significantly influenced overall survival, with survival periods ranging from 14 to 17 months for local recurrence and 10 to 11 months for cats with distant metastases.

Critics may argue that adopting a more complex staging system for feline mammary tumors could increase diagnostic complexity and costs, particularly in resource-limited practices. However, advancements in diagnostic tools and digital pathology may mitigate these challenges. Standardized staging systems offer the potential for improved prognostication and treatment outcomes, as seen in human oncology

This prognostic analysis has limitations, particularly its retrospective nature and the lack of clinical information, which hindered standardized assessment of diagnostic techniques and staging. Treatment decisions, including surgery and adjuvant therapies, were often influenced by guardians’ financial, emotional, and logistical factors. Survival outcomes were also affected by euthanasia decisions, either due to poor prognosis or concerns over chemotherapy’s impact on quality of life. Prospective studies could provide more reliable and comprehensive data, addressing these limitations.

## 5. Conclusions

The findings of this study suggest that the staging system used for women could be beneficial if applied to domestic animals. Ulceration was identified as a significant factor influencing survival outcomes in female cats. Although inflammatory carcinomas were not observed in this study, integrating a T_4_ category into the TNM system remains crucial. Future research should assess lymph node involvement based on the size of the metastasis (including the location, size, and arrangement of neoplastic cells) and/or the number of affected lymph nodes and lymphocenters (distributed ipsilaterally vs. contralaterally). Additionally, establishing standardized reporting guidelines could improve result comparability and foster international interdisciplinary collaboration in veterinary oncology.

## Figures and Tables

**Table 1:** Representation of anatomical groups according to the WHO and the NS system.

WHO Stage		New Staging	
I	T_1_ N_0_ M_0_	I	T_1_ N_0_ M_0_
II	T_2_ N_0_ M_0_	II	T_2_ N_0_ M_0_
III	T_1_ or _2_ N_1_ M_0_ T_3_ N_0_ or N_1_ M_0_	III_A_	T_3_ N_0_ M_0_
		III_B_	T_4_ N_0_ M_0_
		III_C_	Any T N_1_ M_0_
IV	Any T Any N M_1_	IV	Any T Any N M_1_

Abbreviations: T—primary tumor size; N—regional lymph node involvement; M—distant metastases; N_0_—absent; N_1_—present; M_0_—absent; M_1_—present; T_1_—<2 cm; T_2_—2–3 cm; T_3_—>3 cm; T_4_—any size with ulceration. Stage III_A_ (T_3_ N_0_ M_0_)—Tumors >3 cm, without regional lymph node involvement or distant metastasis. Stage III_B_ (T_4_ N_0_ M_0_)—Tumors of any size with direct extension to the chest wall, abdominal wall and/or skin ulceration. This stage also includes inflammatory mammary carcinomas; Stage III_C_ (Any T N_1_ M_0_)—Tumors of any size with regional lymph node metastasis.

**Table 2:** Clinicopathological description of mammary carcinomas.

Clinicopathological Variables		*n* (%)
Number of lesions (*n* = 75)	Single	35 (46.7)
	Multiple	40 (53.3)
Anatomic location (*n* = 71)	Thoracic	23 (32.4)
	Abdominal	29 (40.8)
	Combined	19 (26.8)
Tumor size (cm) (*n* = 75)	T_1_—<2 cm	36 (48.0)
	T_2_—≥2–<3 cm	16 (21.3)
	T_3_—≥3	23 (30.7)
Ulceration (*n* = 75)	Absent	61 (81.3)
	Present	14 (18.7)
Histological type (*n* = 75)	Tubulopapillary C.	43 (57.3)
	Solid C.	15 (20.0)
	Cribriform C.	9 (12.0)
	Micropapillary C.	4 (5.3)
	Others	4 (5.3)
Histological grade (*n* = 75)	I	9 (12.0)
	II	28 (37.3)
	III	38 (50.7)
Lymphovascular invasion (*n* = 75)	Absent	48 (64.0)
	Present	27 (36.0)
Lymph node metastasis (*n* = 75)	Absent	41 (54.7)
	Present	34 (45.3)
Metastasized lymph node (*n* = 34)	Axillary	10 (29.4)
	Superficial inguinal	21 (61.8)
	Both	3 (8.8)
Distant metastasis (*n* = 75)	Absent	73 (97.0)
	Present	2 (3.0)

Abbreviation: C.—Carcinoma

**Table 3:** Analysis and statistical association between variables and disease-free interval and overall survival.

	Disease-Free-Interval (Days)					Overall Survival (Days)				
	*n*	Median (d)	Cumulative Survival (%)		*p*	*n*	Median (d)	Cumulative Survival (%)		*p*
Variables			500 d	1000 d				500 d	1000 d	
Age (years)					0.829					0.427
≤11	38	348	42.2	28.2		41	518	51.9	25.5	
>11	34	318	42.4	***		34	374	34.6	24.3	
Number of lesions					0.524					0.689
Single	34	348	43.3	38.9		35	374	42.1	28.3	
Multiple	38	291	40.7	28.5		40	466	45.9	24.3	
Anatomic Location					0.698					0.714
Thoracic	23	348	43.5	37.3		23	461	49.4	28.2	
Abdominal	27	318	40.4	26.9		29	356	41.4	20.7	
Combined	18	226	33.7	33.7		19	405	32.1	16.0	
Tumor Size (cm)					0.003					<0.001
T_1_ (<2)	36	318	35.3	30.9		36	466	47.8	30.7	
T_2_ (≥2 and <3)	15	***	80.0	62.2		16	890	67.7	40.6	
T_3_ (≥3)	21	129	23.8	15.9		23	141	21.7	5.4	
Ulceration					0.010					0.001
Absent	59	469	46.2	36.5		61	492	49.8	29.0	
Present	13	129	23.1	0		14	141	21.4	0	
Type of surgery					0.160					0.555
Regional	37	296	31.3	22.3		38	374	37.1	15.9	
Unilateral	25	482	49.6	38.6		27	461	48.6	31.9	
Bilateral	10	***	60.0	60.0		10	630	57.1	34.3	
Histological type					0.430					0.437
Tubulopapillary C.	42	348	46.8	40.1		43	466	48.5	27.7	
Micropapillary C.	4	212	25.0	0		4	225	25.0	0	
Solid C.	14	226	26.8	0		15	405	38.1	0	
Cribriform C.	9	296	41.7	***		9	296	41.7	41.7	
Others	3	541	***	0		4	45	50.0	0	
Histological grade					<0.001					<0.001
I	9	***	100	100		9	1500	100	100	
II	28	***	54.9	41.2		28	492	48.4	18.5	
III	35	***	17.3	8.7		38	225	27.6	12.6	
LVI					<0.001					<0.001
Absent	47	606	57.8	45.7		48	678	58.2	39.0	
Present	25	215	11.4	0		27	263	19.8	0	
Lymph node metastasis					<0.001					<0.001
Absent	39	***	67.7	59.2		41	670	58.0	41.3	
Present	33	215	12.8	6.4		34	263	27.7	7.7	
Distant metastasis					0.699					<0.001
Absent	70	344	41.6	32.8		73	461	45.4	25.8	
Present	2	185	50.0	0		2	34	0	0	
WHO staging					<0.001					<0.001
I	22	***	64.8	57.6		23	670	60.8	49.7	
II	10	***	80	***		10	881	67.5	28.1	
III	40	215	20.6	10.3		40	288	31.2	10.3	
IV	n/a	n/a	n/a	n/a		2	34	0	0	
New staging					<0.001					<0.001
I	21	***	62.8	***		21	1031	61.6	55.5	
II	8	***	87.5	***		8	881	71.4	35.7	
III_A_	5	541	60	30		5	691	60	30	
III_B_	5	624	60	0		6	255	33.3	0	
III_C_	33	215	12.8	6.4		33	288	28.5	7.9	
IV	n/a	n/a	n/a	n/a		2	34	0	0	
Adjuvant treatment					0.092					0.612
No	44	624	54.1	46.9		47	361	42.9	34.7	
Yes	28	318	24.1	14.4		28	466	47.0	10.9	
Local recurrence					n/a					0.022
No	n/a	n/a	n/a	n/a		52	492	48.9	35.2	
Yes	n/a	n/a	n/a	n/a		23	356	34.8	6.5	
Distant metastases					n/a					<0.001
No	n/a	n/a	n/a	n/a		33	1031	76.0	57.4	
Yes	n/a	n/a	n/a	n/a		42	257	21.4	3.6	

Note: Abbreviations; n/a—not applicable; d—days; C—carcinoma; M1—clinical or imagological evidence of distant metastasis at the time of diagnosis, to limit the tumor extension; T—primary tumor size; N—regional lymph node involvement; M—distant metastases; N_0_—absent; N_1_—present; M_0_—absent; M_1_—present; T_1_—<2 cm; T_2_—2–3 cm; T_3_—>3 cm; T_4_—any size with ulceration; LVI—lymphovascular invasion. ***—non-computable values (SPSS system information, version 29.0.

**Table 4:** Univariable Cox regression analysis of WHO stage and the new staging system for disease-free interval and overall survival in the 75 cats with mammary carcinomas.

Variable	Disease-Free-Interval			Overall Survival		
	HR	95% CI	*p*	HR	95% CI	*p*
WHO Stage			<0.001			<0.001
I	1		-	1		
II	0.261	0.120–0.570	0.001	1.248	0.458–3.403	0.665
III	0.130	0.031–0.545	0.005	3.087	1.562–6.104	0.001
IV	n/a	n/a	n/a	23.171	4.607–116.553	<0.001
New Stage *			0.001			
I	1		-	1		-
II	0.322	0.04–2.620	0.290	1.118	0.341–3.665	0.854
III_A_	2.080	0.536–8.064	0.289	2.100	0.642–6.873	0.220
III_B_	1.932	0.499–7.473	0.340	4.390	1.526–12.627	0.006
III_C_	4.622	2.007–10.641	<0.001	3.819	1.795–8.125	0.001
IV	n/a	n/a	n/a	28.186	5.442–145.980	<0.001

Note: * Stage I: T_1_ N_0_ M_0_; Stage II: T_2_ N_0_ M_0_; Stage III_A_ (T_3_ N_0_ M_0_)—tumors > 3 cm, without regional lymph node involvement or distant metastasis. Stage III_B_ (T_4_ N_0_ M_0_)—tumors of any size with direct extension to the chest wall, abdominal wall and/or skin ulceration. This stage also includes inflammatory mammary carcinomas; Stage IV Any T Any N M_1_. Abbreviations: n/a—not applicable; CI—confidence interval; HR—hazard ratio.

## Data Availability

The datasets presented in this article are not readily available because the data are part of an ongoing study. Requests to access the datasets should be directed to the corresponding author, fqueirog@utad.pt.

## Abbreviations

The following abbreviations are used in this manuscript:

AJCC	American Joint Committee on Cancer
DFI	Disease-free interval
FMCs	Feline mammary carcinomas
HR	Hazard ratio
III_A_	Stage T_3_N_0_M_0,_ for tumors larger than 3 cm without lymph node involvement
III_B_	Stage T_4_N_0_M_0_, for tumors of any size with direct extension to surrounding tissues (e.g., chest wall, abdominal wall, or skin, including ulceration)
III_C_	Stage III_C_ (Any TN_1_M_0_) for cases with regional lymph node metastases
NS	New staging
OS	Overall survival
T1	Cranial thoracic mammary gland
T2	Caudal thoracic mammary gland
A1	Cranial abdominal mammary gland
A2	Caudal abdominal mammary gland
T_1_	Tumor size <2cm
T_2_	Tumor size 2-3 cm
T_3_	Tumor size >3cm
T_4_	Tumors of any size with direct extension to the chest wall, abdominal wall and/or skin ulceration. This stage also includes inflammatory mammary carcinomas
TNM	Tumor–node–metastasis
WHO	World Health Organization
